# Surgical suture course for dental students with the Peyton-4-step approach versus the PDCA cycle using video assisted self-monitoring

**DOI:** 10.1186/s12903-020-01309-x

**Published:** 2020-12-30

**Authors:** A. Leitmann, Siegmar Reinert, Hannes Weise

**Affiliations:** Department of Oral and Maxillofacial Surgery, University Hospital Tuebingen, Osianderstrasse 2-8, 72076 Tübingen, Germany

**Keywords:** Dental education, CMF-surgery, Curriculum structure, Suturing skills, Video assessment

## Abstract

**Background:**

In this prospective study the Peyton 4-step approach of demonstration–deconstruction–comprehension (verbalization by the learner), and performance by the learner was compared to the PDCA cycle/Deming-Circle (Plan–Do–Check (video assisted self-monitoring)–Act) as a teaching method for surgical suturing and nodes with end performance as the primary objective.

**Methods:**

Students of the third clinical semester in dental medicine were randomly selected to one of the two teaching methods. They completed a first course during the third clinical semester and a subsequent course during the fourth clinical semester. The focus was on learning surgical suturing techniques. Before the course started a questionnaire was handed out to both groups to evaluate their initial level of performance. Each course ended with a practical test to review the content of the course. The evaluation followed standardized parameters. Some of the test tasks in test one were repeated in test two to measure a horizontal as well as vertical difference in performance level.

**Results:**

53 students (Peyton: n = 28/18 female, 10 male; PDCA: n = 25/14 female, 11 male) have completed both courses. The evaluation of the subjective questionnaires showed that the members of the PDCA-groups achieved a higher subjective increase in performance. The objective results also indicated higher learning success in the PDCA-groups compared to the Peyton-Group.

**Discussion/Conclusion:**

This study demonstrated significant learning success for both groups in their own self-assessment as well as in the results of the practical exercises. Subsequently, the superiority of the PDCA cycle could be shown for almost all criteria for surgical suturing techniques. Several studies prioritize the teaching of practical skills according to Peyton and consider step 3 (“comprehension”) to be the essential factor. The PDCA cycle, which has its origins in industrial quality management, and its success can be understood from the perspective of learning theory in terms of Jean Piaget’s model of equilibration. The necessity of active reflection on the learning content through practice constitutes the key element for transfer into long-term memory.

## Background

Treatment of intraoral wounds and bleedings by appropriate suturing techniques is one of the mandatory basic skills of adequate patient care for dentists. Teaching the practical skills for complex techniques like surgical suturing and knot tying can be regarded as especially challenging for the teacher. Teaching needs to be absolutely clear and instilled to ensure that the students can make subsequent use of these skills when their studies have ended.

In all segments of medical education, especially in dentistry, appropriate and sustainable teaching methods and adequate lesson design is of the highest importance for learning motor processes and best repeatability. In medical education, Peyton’s four-step approach has gained in importance in various segments alongside the conventional and frequently used method of “See one, do one, teach one”. Peyton’s approach consists of the following fundamental process steps: Demonstration in real time—deconstruction (single steps are demonstrated and discussed)—comprehension (verbalization by the students, practice performed by the teacher)—performance (the students practice). Section three, ‘comprehension’ is considered the key factor for better learning [1, 2]. So far, studies have only partially demonstrate the superiority of these methods compared to others [1, 3–8].

Another approach, better known from quality management, is the PDCA cycle or Deming circle [9]. The sequence is set out as: Plan–Do–Check–Act. The third step “check” is here also suspected the key element for successful teaching in this method, too. In contrast to the method according to Peyton, when the students are practising, they learn from their own mistakes by self-verification and through assistance—in this study, this was implemented by video-assisted self-monitoring (Additional file 1).

This prospective study aimed to determine the best applicable method for teaching practical skills for surgical suturing and knot tying for dental students at the Eberhard Karls University Tuebingen.

The objective of this study was to test the hypothesis that the common teaching method according to Peyton is superior to the PDCA cycle.

## Methods

All dental students of the third clinical semester were split into groups of two students each during the winter semester 2016/2017 and the summer semester 2018. These groups of two were randomly assigned to either the teaching method according to “Peyton” or the “PDCA—cycle”. Each group of two completed during the whole time of the study two courses in two structures modules of the allocated teaching method, the first course during the third clinical semester, the second course during the fourth clinical semester. These courses were always held immediately prior to the practical surgery weeks at the centre for Dentistry, Oral Medicine and Maxillofacial Surgery within the University Hospital Tuebingen, which are already an integral part of the curriculum. The objective of the courses is to enable students to transfer the skills achieved on the surgical suturing dummy directly to the patient.

Course attendance was mandatory to prepare the students for hands-on patient treatment and for their upcoming state examinations, whereby participation in the study was voluntary and was anonymized.

All courses were delivered by the same teacher. The course content was equal in both groups. The focus of the first course was to practice single stitch sutures as well as the correct and reliable mastery of the surgical knot using the needle holder and the surgical hand knot using both hands.

**Fig. 1:**
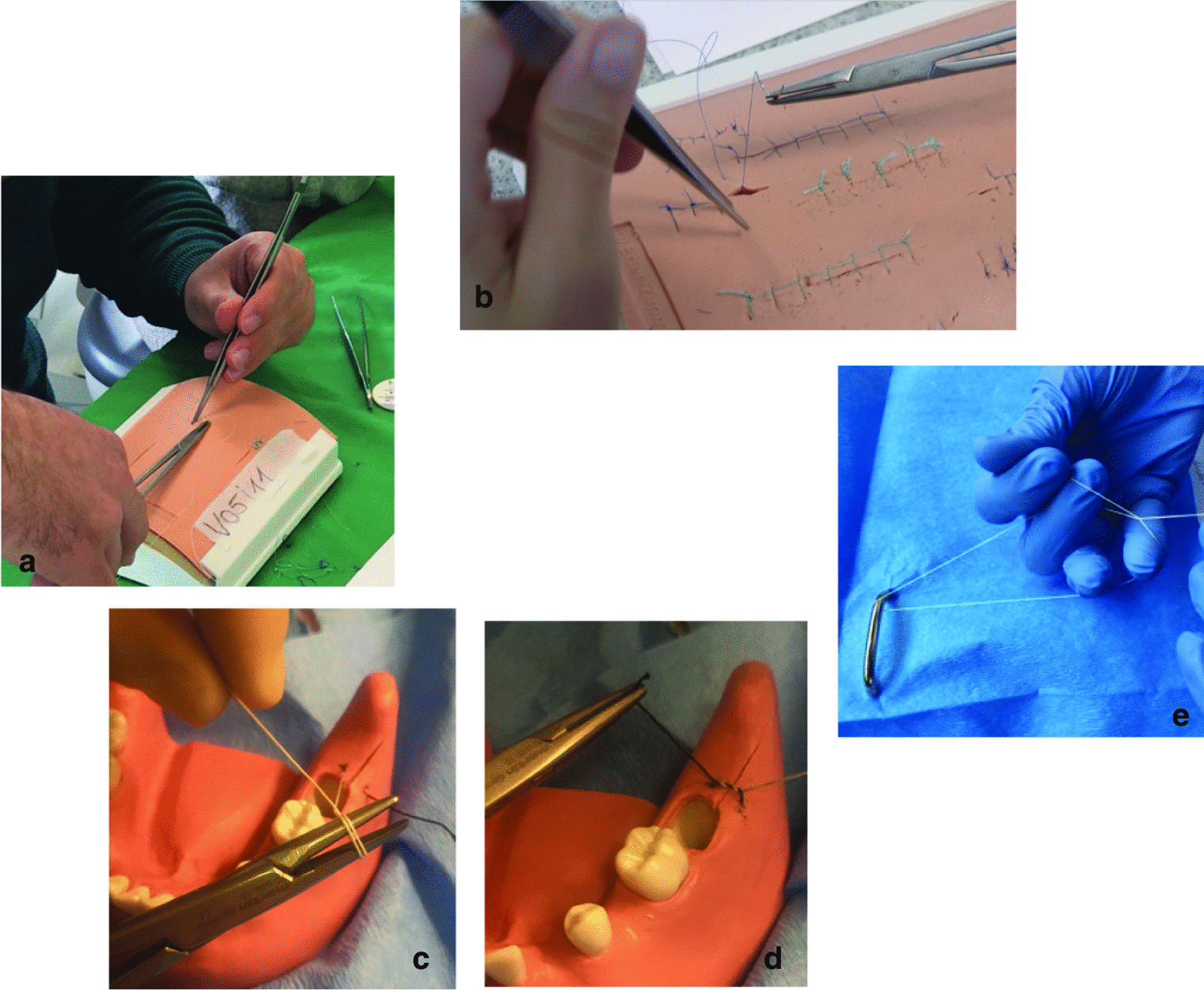
Photo documentation of the students during the course. **a** Interrupted suture during stress test. **b** Exercises with artificial skin. **c**, **d** Interrupted suture on pine model. **e** Practice of manual knotting techniques

During the second course, this content was repeated leaving each student to practice at their own pace. The course also included additional suture techniques such as the sequential Reverdin suture and the wreath suture (Fig. 1).

**Fig. 2:**
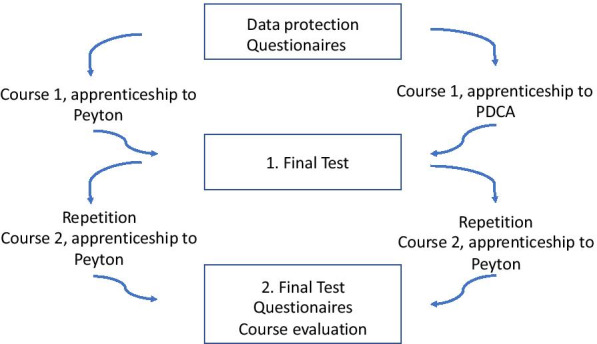
Study procedure

To compare the results of both teaching methods, a subjective questionnaire was handed over to both groups prior to the first course and after completion of the second course (Fig. 2) to verify the process of self-assessment and safe operation. Furthermore, a short practical exam was conducted after each course.

The task for the students was to close a given wound using a specific suture technique (such as single knot, continuous Reverdin-suture) on time and to fix the threads using a surgical knot. During the tests, the suture trainer was marked with a pseudonym and the hands of the students were filmed to obtain an exact rating of individual performance. Amongst other skills, the correct use of the needle holder and scissors, the atraumatic treatment of wound edges, uniform distance of the individual sutures to each other and to the wound and the correct suture direction of the hand knots were rated.

Some of the test tasks of test one were repeated in test two, thus creating not only the possibility of horizontal comparisons of the results between the two teaching methods, but also the vertical measurement of the student´s increase of performance from course 1 to course 2.

For the Peyton group, the course started with a 20 min Powerpoint presentation to convey background theoretical knowledge of suturing techniques and knots. In addition, each action was preceded by a demo video played in real time, which was then broken down into individual steps and discussed. After this theoretical preparation, the teachers repeated the instructions directly to the students whereby the procedure was again explained step-by-step. The students were then given 45 min for practical exercises.

For the PDCA groups, the lesson started with the Powerpoint presentation similar to the Peyton groups but instead of demo videos the students were presented with photos showing each step in the suture technique. These groups were also given 45 min to practice. During the exercises, the students were filmed in short sequences during the exercises. These video clips were then presented on a widescreen monitor in the class room followed by a discussion.

We confirm that we have read the Helsinki Declaration and have followed the guidelines in this investigation. This study has been approved by the local ethical committee.

### Statistical analysis

All results were gathered and sorted in Excel files. For calculation the software IBM SPSS Statistic 24 was used with a significance level/*p* value if 5% for all applied tests (chi-quadrat, *t* test and cross-tabulation). Statistical analysis were verified by the Institution for clinical epidemiology and applied biometry.

## Results

53 students took part in the study. 28 students (14 couples; 18 female, 10 male) completed the two interrelated courses (third and fourth clinical semester) according to the teaching method of Peyton, 25 students (11 couples, one three-men group; 14 female, 11 male) completed the two courses according to the teaching method of PDCA-Cycle.

The evaluation of the subjective questionnaires showed that the members of the PDCA-groups achieved a higher subjective increase in performance.

For example, before the courses an average of 81% of the PDCA-group did not feel confident enough to close a skin incision, smaller intraoral wounds, bleeding lacerations or a tooth extraction site by interrupted suture. After finishing both courses, an average of 76% of the course participants stated that they were now well or very well prepared to perform these techniques. For the same categories, the Peyton groups had an increase of performance skills of 65% (I am not capable) at the beginning of the courses, to 71% (I can do that (very) well now) after finishing both, courses which indicates a significant lower subjective increase in performance.

**Fig. 3:**
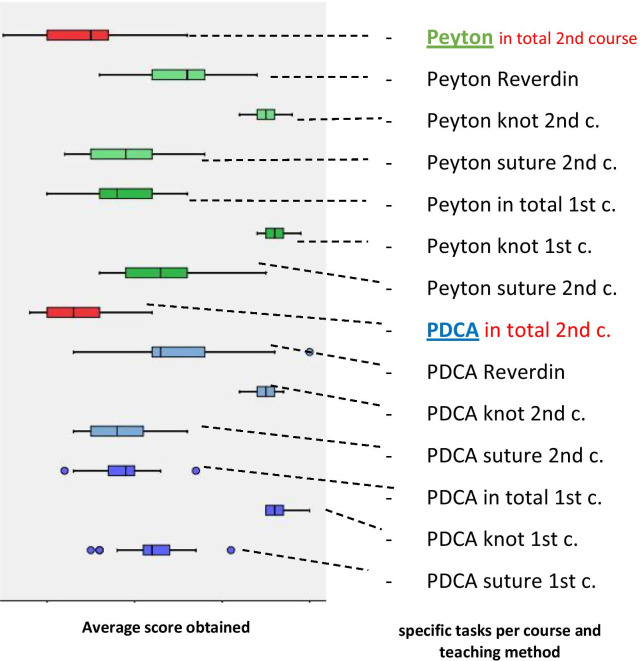
Students test results for the individual tasks on average, classified according to the teaching method and course 1 (third semester) to course 2 (fourth semester) (blue: PDCA, green: Peyton, red: total results). You can see, that the results in Peyton-groups do have a wider range compared to results of the PDCA-groups and that PDCA-groups reached on average a few points more than Peyton-groups in every single specific task—except the knot-task in first courses

The objective results also indicated higher learning success in the PDCA-groups (Fig. 3).

Comparing the results, after finishing the first course, the PDCA-group achieved better results in the tests (34 points max.), i.e. on average 0.17 points more. This was also the case after completion of the second course (57 points max.) in all individual tests, i.e. on average 0.69 points more than the Peyton group. Furthermore, comparing the repeated tasks of test two taken from test one, the difference in the achieved score, i.e. the objective increase of performance of the PDCA-group, was higher (d-Peyton = 4.4; d-PDCA = 4.92).

The “knot first course” was the only subtask of in total 12 subtasks where the Peyton groups achieved a higher score level (Fig. 3).

Hence the PDCA groups achieved a higher score level in the remaining 11 subtasks as well as in the average overall result.

Due to the low number of participants, none of the results where statistically significant (*p* > 0.05%).

## Discussion

So far, the increasing popularity of the teaching method described by Peyton can be attributed simply to the positive feedback of the students, who often feel better supported by a more elaborate lesson design in terms of graphical and interactive presentation than is the case in conventional teaching methods. This is also the conclusion of the study by Hanson et al. [10] who found that video footage is especially well accepted by the students and will, if available, be used for repetition of the subject material after course completion. The positive results of other studies that verify teaching success have motivated lecturers to make use of this teaching method [1, 5, 6, 11].

It can be said that we achieved a consistently positive overall result for both groups in this study, which leads to the assumption that any lessons that are diligently prepared and presented in an interesting and interactive way will increase student attentiveness and their readiness to learn in contrast to conventional classroom teaching and all too brief instructions.

Several didactic approaches were integrated simultaneously into the PDCA cycle as well as into the teaching method by Peyton. For example, Jawhari et al. describes Peyton’s steps 1 and 2 as “the perceptually processed information” which in Peyton’s step 3 is “actively manipulated within the working memory to transfer the information into the long-term memory” 1.

The evaluation of our own data has shown that teaching based on the PDCA cycle using video-assisted self-monitoring at almost every step was superior to the Peyton group. This was confirmed by the scores on the subjective questionnaires and by the objective test results. The differences between the two teaching methods can be identified as the slightly longer practice time given to the PDCA group (practice started immediately after the presentation) and increased student attentiveness for verbal communication of the correct procedure via video, especially when the students were shown video feedback of themselves. Furthermore analysing the failures of all groupmembers gives a positive effect on own performance. Learning theory also provides evidence for the success of the PDCA cycle. Dewey et al. already stated the need to reflect on your own actions or experiences as an important factor for transfer into long-term memory. According to this theory, the occurrence of a problem during an action and the search for possible causes and a solution strategy (for example: Why is my knot not holding?) triggers a sustainable learning process that is reinforced by adequate follow up, e.g. by talking through the video sequences.

The constructivist approach, especially according to the model of equilibration by Piaget, supports this theory. Direct subjective experience of objective reality, here the subject matter to be learned, forms the basic building block of the learning process. The main emphasis is placed on intersubjective communication, i.e. mutual discussion of the new material [12].

In an effort to assign the PDCA-cycle to one of the existing and well-known teaching strategies, the closest approach would be “problem-oriented learning”. According to this teaching method, the focus is on stimulating the long-term memory which should guarantee learning success, especially after finishing the period of study. In this approach the students, in contrast to didactic teaching, are able to concentrate their learning efforts in line with their existing strengths and weaknesses [13, 14].

In direct comparison of the two teaching methods in the context of this study, the differences are quite low in the objective tests. This might be explained by the very elaborated concept of both teaching methods and the lessons being given to very small groups. The advantage of a small group is, that regardless of the teaching method, the instructor can give more time and attention to the individual students.

Nonetheless, the superiority of the PDCA cycle was found to be significant in a total of 11 out of 12 subtasks compared to the Peyton method.

Performance increase in the PDCA-group was also found to be higher during the course of the study and students’ results are were clearly more homogeneous (Fig. 2).

## Limitations

For limitations of this study we have to point to the small number of study participants so far, due to the small semester-classes. We plan to continue the study for several years, so that we can make a second analysis with more participants in some time.

Furthermore none of our results showed statistically significance and the differences are quite low in the objective tests. Surely we would have had a bigger difference if the teaching methods would distinguish more. With a higher number of participants we hope to get more clear results on this spot.

## Conclusion

Overall, in our setting for teaching the practical skills of “surgical suturing and knot techniques” to students of dentistry, the PDCA cycle was found to be superior for almost all evaluation criteria. However, the results were not significant for any of the criteria.

Based on our results, we have decided to implement the PDCA cycle method in student lessons, however we will continue to verify the results with regard to their significance based on further test subjects.

There is no doubt that the positive overall ratings of the courses and the excellent results achieved in both groups provide the impetus to continue the refinement of university teaching, especially for courses with a very practical component such as dentistry, through interactive lesson design lessons and by utilizing all relevant media to achieve best possible knowledge transfer.

## Supplementary information

**Additional file 1.** Peyton’s four-step approach: differential effects of single instructional steps on procedural and memory performance–a clarification study. Annual meeting of the German Association for Medical Education.

## Data Availability

All data and materials are accessible on a local server of the Department of Oral and Maxillofacial Surgery of the University Hospital Germany.
