# Supplementary material for: Surgical suture course for dental students with the Peyton-4-step approach versus the PDCA cycle using video assisted self-monitoring
Source: BMC Oral Health. 2020 Dec 30;20:365. doi: 10.1186/s12903-020-01309-x (PMC7772909; doi:10.1186/s12903-020-01309-x)
Supplement: Supplementary file 1 — Additional file 1. Peyton’s four-step approach: differential effects of single instructional steps on procedural and memory performance–a clarification study. Annual meeting of the German Association for Medical Education. [file 12903_2020_1309_MOESM1_ESM.docx]

Stress Test

Please complete the following tasks in the time allowed.

In doing so, call to mind the criteria presented in the course on suturing for good functional and cosmetic outcomes (e.g. symmetrical, thread not cutting into the skin)

Evaluation will be by pseudonym.

The results of this test are part of the study and they have no effect on your other study achievements/grades/ etc.

1. Suture a 5 cm cut with 5 single button sutures in 3 minutes.

2. Tie the two threads using the surgical hand knot that you learned in 2 minutes.

Stress Test Evaluation Form

1. Wound suture

Task completed in the time allowed

Correct type of suture

Correct number of sutures

Knots correctly positioned

Correct use of the needle holder and foreceps

Wound adequately closed

Atraumatic handling of the wound edges

Wound margins well aligned throughout

Correct number of button sutures

Uniform distance of the insertion/exit sites to the wound margin

Uniform distance between the single sutures

Total score:

2) Surgical hand knots

Correct knot tying technique

Correct number of knots

Correct sequence, suture direction in general and within the wound

Knots appropriately tensioned

Total score:

Please enter your individual code number as follows::

First letter of your mother’s first name, your birth month as a two-digit number, last letter of your family name, birth month of your father as a two-digit number..

Example: C11N07

This ensures that you remain anonymous. It also means that the course managers can match the pre- and post-evaluations to each other.

General:

Sex m f

Specialization semester

University semester

First degree yes no

If no, my first degree:

I completed vocational training before my studies

yes no discontinued

if yes / discontinued:

My future career goal

Dentist, Oral Surgeon, Orthodontist, maxillo-facial surgeon

I already took one/several extracurricular suturing courses

yes, by MLP, yes, at the Doclab, yes, other, no

if yes, which other course:

Your personal opinion is important

totally agree

agree

disagree

totally disagree

no response

This course is superfluous,

Our curriculum already has enough practical courses …

We already had adequate instruction and practice in suturing.

We have too many practical courses mainly in the areas of ….: :

I would like more practical exercises in suturing

I would like more practical exercises in the areas of …. :

I think I am good at suturing.

I think good suture technique is important for my future career.

I feel confident and able when treating patients.

I feel sufficiently supported by the instructors when treating patients.

How many tooth extractions have you already done independently?

How many intraoral suturing procedures have you already done independently?

I am confident that I have the medical knowledge and clinical skills …

… to close a skin incision with single button sutures and achieve good functional and cosmetic outcomes.

… to close a skin incision with a continuous suture (e.g. Reverdin) and achieve good functional and cosmetic outcomes.

… to suture small intraoral lesions.

…to decide which suturing technique is best in different situations.

… to remove sutures.

… to stop minor intraoral bleeds by appropriate suturing.

… to suture skin grafts and regional and mucoperiosteal flaps, e.g. after tooth extraction.

… to adequately attach a tissue flap by interrupted suturing.

… to extract a tooth.

… to perform gingivectomy.

… to perform and document a specific intraoral examination, and to draw diagnostic, preventive and therapeutic conclusions.

I am anxious about invasive procedures on patients (e.g. scalpel cuts, injections).

I have adequate anatomical and physiological knowledge to perform invasive procedures on patients with confidence.

E.g. I know the different incision patterns, when and where manipulation is contraindicated and where block anaesthesia is placed ….

The course has provided me with a structured approach to adequate suturing.

In the course I learned a sufficient number of suturing techniques with their advantages and disadvantages for me to work confidently as a dentist.

I benefitted from the course.

The learning objectives of the course were clearly defined.

I consider the course methodology helpful for my degree in dentistry.

The course has given me confidence for my future career as a dentist practising oral surgery.

The design of the study material was clear.

The study material was helpful.

The course managers explained the tasks clearly.

Feedback was constructive.
